# Decreased Deposition of Beta-Amyloid 1-38 and Increased Deposition of Beta-Amyloid 1-42 in Brain Tissue of Presenilin-1 E280A Familial Alzheimer’s Disease Patients

**DOI:** 10.3389/fnagi.2020.00220

**Published:** 2020-07-28

**Authors:** Felix Dinkel, Diana Trujillo-Rodriguez, Andres Villegas, Johannes Streffer, Marc Mercken, Francisco Lopera, Markus Glatzel, Diego Sepulveda-Falla

**Affiliations:** ^1^Institute of Neuropathology, University Medical Center Hamburg-Eppendorf – UKE, Hamburg, Germany; ^2^Genetics Institute, National University of Colombia, Bogotá, Colombia; ^3^Neuroscience Group of Antioquia, Faculty of Medicine, University of Antioquia, Medellín, Colombia; ^4^Johnson & Johnson Pharmaceutical Research and Development, Janssen Pharmaceutica, Beerse, Belgium

**Keywords:** beta-amyloid, Presenilin-1, gamma Secretase, familial Alzheimer’s Disease, immunohistochemistry, tau

## Abstract

Familial Alzheimer’s Disease (FAD) caused by Presenilin-1 (PS1) mutations is characterized by early onset, cognitive impairment, and dementia. Impaired gamma secretase function favors production of longer beta-amyloid species in PS1 FAD. The PS1 E280A mutation is the largest FAD kindred under study. Here, we studied beta-amyloid deposits in PS1 E280A FAD brains in comparison to sporadic Alzheimer’s disease (SAD). We analyzed cortices and cerebellum from 10 FAD and 10 SAD brains using immunohistochemistry to determine total beta-amyloid, hyperphosphorylated tau (pTau), and specific beta-amyloid peptides 1-38, 1-40, 1-42, and 1-43. Additionally, we studied beta-amyloid subspecies by ELISA, and vessel pathology was detected with beta-amyloid 1-42 and truncated pyroglutamylated beta-amyloid antibodies. There were no significant differences in total beta-amyloid signal between SAD and FAD. Beta-amyloid 1-38 and 1-43 loads were increased, and 1-42 loads were decreased in frontal cortices of SAD when compared to FAD. Beta-amyloid species assessment by ELISA resembled our findings by immunohistochemical analysis. Differences in beta-amyloid 1-38 and 1-42 levels between SAD and FAD were evidenced by using beta-amyloid length-specific antibodies, reflecting a gamma secretase-dependent shift in beta-amyloid processing in FAD cases. The use of beta-amyloid length-specific antibodies for postmortem assessment of beta-amyloid pathology can differentiate between SAD and PS1 FAD cases and it can be useful for identification of SAD cases potentially affected with gamma secretase dysfunction.

## Introduction

Alzheimer’s disease (AD) is the most common form of dementia in the elderly. The prevalence is estimated to be about 24 to 35 million people worldwide (Reitz and Mayeux, 2014). Clinically, it is characterized by progressive cognitive impairment and behavioral changes, which eventually lead to dementia and death (Giaccone et al., 2011). Neuropathological hallmarks are brain atrophy, beta-amyloid (Aβ) deposits forming plaques in the extracellular space and the accumulation of intraneuronal hyperphosphorylated tau protein (pTau) as neurofibrillary tangles (NFTs) (Alzheimer’s Association, 2010). Age is one of the main risk factors for developing AD (Berr et al., 2005). AD can be divided into two groups: early onset AD (EOAD) occurring before 65 years of age, while 95% of all AD cases are late onset AD (LOAD) and occur after 65 years of age (Alzheimer’s Association, 2010). The presence of allele 4 of Apolipoprotein E (ApoE4) is the main genetic risk factor for LOAD; ∼40% of these patients show at least one ApoE4 allele (Myers et al., 1996; Bettens et al., 2013).

Near to 70% of EOAD are associated with mutations in genes encoding for Amyloid Precursor Protein (APP), Presenilin-1 (PS1), or Presenilin-2 (PS2) (Bertram et al., 2010). More than 190 PS1 pathogenic mutations are the most common cause of familial EOAD (FAD) (Cruts et al., 2012; De Strooper et al., 2012). PS1 is part of the gamma secretase (γ secretase) protein complex, a type 1 intramembrane cleaving protease (Wolfe, 2007; De Strooper et al., 2012) responsible for intramembrane processing of various proteins, including APP (Wakabayashi and De Strooper, 2008; McCarthy et al., 2009). γ secretase acts together with beta secretase in the amyloidogenic processing of APP (De Strooper and Annaert, 2010), and γ secretase sequentially cleaves it into the 38 to 43 amino acids Aβ peptide (Chávez-Gutiérrez et al., 2012). Aβ aggregate in the extracellular space into fibrils and finally form plaques. Mutated PS1 FAD cases show an elevated amount of Aβ 1-42 and increased Aβ 1-42/1-40 ratio (Larner, 2013). Aβ 1-42 is more prone to aggregate than Aβ 1-40 due to its higher hydrophobicity (Wolfe, 2007; Fernandez et al., 2014). Longer Aβ species, particularly Aβ 1-43, have been reported as being more toxic *in vivo*, contributing to neurodegeneration in AD (Burnouf et al., 2015), and CSF assessment of Aβ species ratios provides increased diagnostic value together with Amyloid PET studies (Adamczuk et al., 2015). Besides the variable length, Aβ goes through posttranslational modifications such as the formation of N-terminal pyroglutamates (pE) of truncated Aβ species (Cabrera et al., 2018) with direct impact on Aβ pathogenicity (Sofola-Adesakin et al., 2016). The neuropathological phenotype of PS1 FAD mutations is diverse, including abundant Aβ 1-42 and pTau deposits, prominent atrophy, and neuronal loss. Some PS1 mutations show large diffuse Aβ 1-42-rich cotton wool plaques (Shepherd et al., 2009); others show cerebellar amyloid deposits (Lemere et al., 1996).

Clinically, PS1 FAD is characterized by variable yet early disease onset and full penetrance. Symptoms might include myoclonus, seizures or epileptic syndromes, extrapyramidal symptoms, behavioral and psychiatric features, spastic paraparesis, language impairment, and cerebellar ataxia. Neuropathologically, aside from typical AD features, some PS1 mutations show cotton wool plaques, cerebral amyloid angiopathy (CAA), Lewy bodies, cerebellar Aβ deposition, and Pick bodies (Larner, 2013). In Antioquia, Colombia, a large kindred of FAD with a PS1-E280A mutation has shown early onset of disease, language difficulties, cerebellar ataxia, seizures, and myoclonus (Sepulveda-Falla et al., 2012). Neuropathological studies have shown faster rates of NFTs formation (Gómez-Isla et al., 1999), high neuronal loss, and increased Aβ 1-42 levels with cerebellar pathology (Shepherd et al., 2009), specific pTau aggregation in the cerebellum (Sepulveda-Falla et al., 2011), and Purkinje cell loss (Sepulveda-Falla et al., 2014).

Given that abnormal Aβ processing into longer Aβ species is a known feature in PS1 FAD (Chávez-Gutiérrez et al., 2012) and in PS1 E280A (Van Vickle et al., 2008), we studied morphology and distribution profiles of different Aβ species in brain regions of PS1 E280A FAD patients to make a direct comparison with SAD brain tissue. We found a specific Aβ 1-38/1-42 immunohistochemical profile associated with FAD when compared with SAD and decreased Aβ 1-42/3pE-x ratio of affected vessels in FAD.

## Materials and Methods

### Patients and Human Brain Samples

Presenilin-1 E280A genealogy was identified 30 years ago and mutation carriers have been followed up since then. Carriers for the present study were identified and affected patients went through neurological and neuropsychological characterization and were followed up using the CERAD protocol, NINCDS-ARDA, and DSM-IV criteria until end-stage dementia and death (Acosta-Baena et al., 2011; Sepulveda-Falla et al., 2011). For neuropathological studies, we collected postmortem brain tissue of 10 PS1-E280A patients and matched them with 10 SAD patients (Table 1) showing corresponding CERAD and Braak stages (Braak and Braak, 1991; Gearing et al., 1995) (neuropathological analysis were performed by MG and DSF, Table 1). Brain donation and procedures were performed following ethical approval from respective institutions (Universidad de Antioquia, UKE). Formalin-fixed samples from frontal, temporal, parietal, occipital cortex, and cerebellum were selected for morphological studies. Further studies of Aβ species were conducted in frontal cortices.

**TABLE 1:** Demographic profile of studied cases.

**Group**	**Case**	**Gender**	**Age of Onset**	**Age of Death**	**Postmortem (h)**	**Brain Weight (g)**	**Braak**	**CERAD score**	**ApoE**
PS1-E280A FAD	1	F	47	54	5.5	1029.0	VI	C	3/3
PS1-E280A FAD	2	M	44	52	4.8	1061.3	VI	C	3/3
PS1-E280A FAD	3	M	47	56	3.3	941.6	VI	C	3/3
PS1-E280A FAD	4	F	46	66	4.0	581.5	VI	C	3/4
PS1-E280A FAD	5	F	49	62	4.0	968.7	VI	C	3/3
PS1-E280A FAD	6	F	48	54	3.0	651.7	VI	C	3/3
PS1-E280A FAD	7	M	49	55	2.8	980.5	VI	C	4/4
PS1-E280A FAD	8	F	50	60	2.8	768.1	VI	C	3/3
PS1-E280A FAD	9	F	52	68	6.4	763.3	VI	C	3/3
PS1-E280A FAD	10	M	47	58	3.5	937.2	VI	C	3/3
Mean/% (SD)		60%	47.9 (2.2)	58.5 (5.4)	4.0 (1.2)	868.3 (165.4)	100%	100%	20%
SAD	11	M	ND	67	10	1225.0	V	C	3/3
SAD	12	M	80	86	6.3	1200.0	IV	C	ND
SAD	13	F	55	70	11.3	890.0	IV	B	3/4
SAD	14	F	79	87	2.8	842.8	V	C	3/4
SAD	15	F	82	91	4.5	956.0	VI	C	3/3
SAD	16	F	65	74	2.5	846,0	IV	C	3/3
SAD	17	F	65	76	4.3	ND	V	C	4/4
SAD	18	F	69	76	8.0	ND	VI	C	3/4
SAD	19	M	70	83	4.3	981.1	VI	C	3/2
SAD	20	F	50	61	4.1	ND	VI	C	3/3
Mean/% (SD)		70%	68.3 (11.0)	77.1 (9.6)	5.81 (3.0)	991.5	40%	90%	40%
*t* test/c^2^ value		0.22	−3.56	−3.48	−1.36	−1.07	8.57	1.05	1.31
*p*		1.000	0.000***	0.001***	0.173	0.283	0.003**	1.000	0.252

### Histopathological Methods

All morphological analyses were performed on 3-μm-thick deparaffinized sections. Immunohistochemical stainings were performed following pre-treatment for antigen retrieval and probed with monoclonal anti-Aβ domain antibody (6E10,1:100, Covance, Maidenhead, United Kingdom) and anti pTau (pSer202 and pThr205) antibody (AT8, 1:1500, Thermo Scientific, Rockford, IL, United States). The following purified monoclonal antibodies against different Aβ species were used: Aβ x-38 (J&JPRD/Aβ38/5, 1:2000, Janssen Research & Developement, Beerse, Belgium), Aβ x-40 (JRF/cAβ40/28, 1:1000, Janssen Research & Developement, Beerse, Belgium), Aβx-42 (JRF/cAβ42/26, 1:1000, Janssen Research & Developement, Beerse, Belgium), Aβ 1-43 (1:1000, Genetex, Irvine, CA, United States), and AβN3pE-x (J&JPRDAβ/pE3/1, 1:1000, Janssen Research & Developement, Beerse, Belgium). Stainings were performed on an automated Ventana HX system (Ventana Medical Systems, Tucson, AZ, United States) following the manufacturer’s instructions. Experimental groups were stained in one run for each antibody in order to provide uniform staining conditions. Primary antibodies were visualized using a standard diaminobenzidine streptavidin-biotin horseradish peroxidase method (Sigma Aldrich, Hamburg, Germany). Amyloid plaques were assessed and classified according to standard criteria (Duyckaerts et al., 2009). For quantification of primary antibodies immunosignal, three representative regions (0.1349 mm^2^ each) were analyzed by quantifying the area immunoreactive for Aβ as a percentage of the total area for each image using the AxioVision 4.6 software (Carl Zeiss, Oberkochen, Germany) according to published methods (Debatin et al., 2008). We evaluated cerebral amyloid angiopathy (CAA) in SAD and FAD cases alongside the Vonsattel grading scale (Vonsattel et al., 1991), as the percentage of vessels identified with more than two thirds of their perimeter immunostained for specific Aβ antibodies in the frontal cortex. Twenty vessels were evaluated per patient.

### Biochemical Methods

Solid phase sandwich enzyme-linked immunosorbent assay (ELISA) for Aβ 1-40 and Aβ 1-42 (Invitrogen, Camarillo, CA, United States) and that for Aβ 1-38 and Aβ 1-43 (IBL, Hamburg, Germany) were used according to manufacturer protocols. Briefly, 100 mg of snap-frozen tissue from frontal cortices were homogenized in 800 ml of 5 M guanidine HCl/50 mM Tris–HCl for Aβ solubilization as reported (Selkoe, 1994; Cotman, 1997). Homogenates were mixed for 4 h at room temperature, diluted in phosphate-buffered saline/5% bovine serum albumin/0.03% Tween 20, and centrifuged at 16,000 × *g* for 20 min at 4°C. Supernatant was collected and probed with the ELISA kit for each antigen. Samples were measured at 450 nm in a BioTek mQuant spectrophotometer (Winooski, VT, United States) and expressed as pmol/mg of total protein.

### Statistical Analysis

Data were analyzed using IBM SPSS Statistics 22 software (IBM/SPSS Inc., Armonk, NY, United States) and GraphPad Prism 6 (GraphPad Software, Inc., La Jolla, CA, United States). Analyses included Kolmogorov–Smirnov and Shapiro–Wilk tests for normal distribution assessment. For non-parametric comparisons, we applied Mann–Whitney *U* (given as *Z*) for two group comparisons and Chi square test was applied to categorical variables. Correlation analysis was performed using Spearman Rho’s test. Statistical significance of all analyses was determined with ^∗^*p* ≤ 0.05, ^∗∗^*p* ≤ 0.01, and ^∗∗∗^*p* ≤ 0.001.

## Results

### Similar Morphology and Distribution of 6E10 Detected Aβ Pathology in SAD and PS1 E280A FAD

**FIGURE 1:**
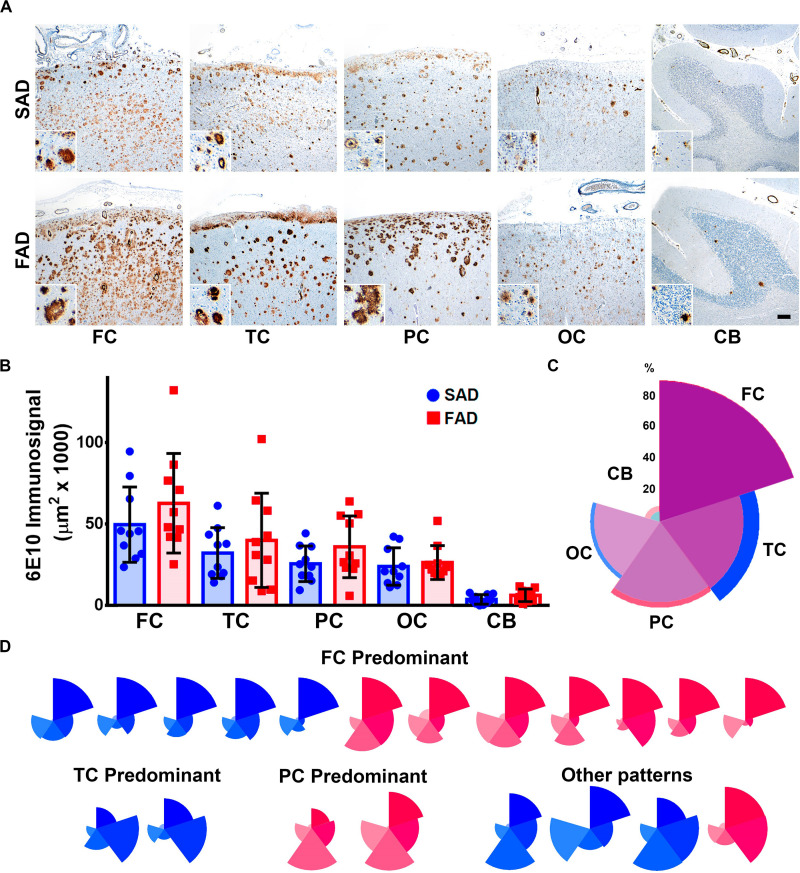
Distribution of Aβ pathology in brain regions of SAD vs. FAD PS1 E280A. **(A)** Immunohistochemical staining for total Aβ with 6E10 in frontal cortex (FC), temporal cortex (TC), parietal cortex (PC), occipital cortex (OC), and cerebellum in patients with SAD and FAD (scale bar = 200 mm). Insets depict Aβ deposits at higher magnification for each region (scale bar = 70 mm). **(B)** Quantification of Aβ immunosignal present in frontal cortex, temporal cortex, parietal cortex, occipital cortex, and cerebellum. No statistically significant differences in all evaluated areas were observed between SAD and FAD cases. **(C)** Radar chart for the 6E10 percentage of immunosignal distribution according to brain regions in SAD (blue) and FAD (red) cases. Both groups present a FC-predominant distribution pattern. **(D)** Radar charts for individual 6E10 immunosignal distribution pattern for all SAD (blue) and FAD (red) cases studied. FC predominant distribution pattern was identified among both groups, with seven FAD cases and five SAD cases. TC and PC patterns were identified in SAD and FAD cases, respectively, with two cases for each. Some cases presented dissimilar patterns in both groups, with three SAD cases and one FAD case.

Detected Aβ immunosignal in FAD did not differ significantly from SAD in all five brain regions studied (Figures 1A,B). Both SAD and FAD cases showed diffuse, core, and neuritic Aβ plaques. FAD cases present with abundant diffuse plaques, as previously described (Shepherd et al., 2009). SAD cases also showed abundant diffuse plaques, particularly in frontal and temporal cortices. There was high variability in the size and distribution of plaques in both groups and studied group regions (Figure 1A). The highest average immunosignal was detected in the frontal cortex of FAD as well as of SAD patients, followed by temporal cortex, parietal cortex, and occipital cortex, with the lowest Aβ signal being detected in the cerebellum (Figure 1B and Table 2), creating a predominantly frontal aggregation pattern (Figure 1C). When individual aggregation patterns were analyzed, we found that 5/10 SAD and 7/10 FAD cases showed frontal cortex predominance. Two out of 10 SAD showed temporal cortex predominance while 2/10 FAD showed parietal cortex predominance (Figure 1D).

**TABLE 2:** Comparative analysis for Aβ peptides and pTau immunosignal.

** Variable**	**PS1-E280A FAD**		**SAD**		**Value**	***p***
	**Mean**	**SD**	**Mean**	**SD**		
**6E10 Immunosignal Area (mm^2^)**						
Frontal Cortex	56,092.62	7305.29	47,757.28	8995.46	−1.134	0.257
Temporal Cortex	32,339.89	7551.81	36,753.58	8307.88	−0.302	0.762
Parietal Cortex	34,300.06	7083.65	20,753.3	4648.34	−1.285	0.199
Occipital Cortex	25,884.29	3837.6	22,181.8	5235.15	−0.907	0.364
Cerebellum	6235.14	1410.49	3619.2	1351.57	−1.285	0.199
**AT8 Immunosignal Area (mm^2^)**						
Frontal Cortex	182,242.41	15,791.27	96,201.68	18,382.56	−1.89	0.059
Temporal Cortex	175,241.61	14,731.02	150,853.68	11,620.95	−0.756	0.450
Parietal Cortex	175,502.72	21,045.91	120,702.06	22,280.09	−1.663	0.096
Occipital Cortex	202,518.61	15,726.26	145,855.3	15,004.9	−2.041	0,041*
Cerebellum	178.99	59.29	36.62	19.85	−2.117	0,034*
**Immunosignal Area (mm^2^) in Frontal Cortex**						
Aβ 1-38	133,778.76	59,511.88	685,023.95	71,017.07	−3.326	0,001**
Aβ 1-40	98,008.09	12,188.78	128,683.57	53,115.09	−0.907	0.364
Aβ 1-42	199,059.23	16,922.21	160,329.22	22,249.32	−2.268	0,023*
Aβ 1-43	37,831.11	8134.32	305,449.17	136,139.17	−2.343	0,019*
Pyroglutamylated Aβ 3pE-x	321,872.71	34,202.97	306,876.82	60,287.35	−0.454	0.65
**Area Ratios in Frontal Cortex**						
Aβ 1-38/1-42	0.65	0.25	4.58	0.84	−3.628	0,0001***
Aβ 1-42/1-40	2.19	0.27	2.29	0.67	−0.151	0.880
Aβ 1-40/1-43	3.35	0.68	2.3	2.07	−2.797	0,005**
**ELISA values in Frontal Cortex (pmol/g)**						
Aβ 1-38	22.21	8.59	52.67	31.83	0.000	1.000
Aβ 1-40	121.16	58.28	113.56	71.21	−0.227	0.821
Aβ 1-42	522.21	147.28	161.01	40.32	−2.57	0,01**
Aβ 1-43	9.1	4.93	2.67	1.01	−1.739	0.082
**ELISA Ratios in Frontal Cortex**						
Aβ 1-38/1-42	0.05	0.01	0.41	0.28	−1.361	0.174
Aβ 1-42/1-40	11.05	4.74	6.28	4.22	−1.587	0.112
Aβ 1-40/1-43	24.01	10.33	60.95	28.73	−1.739	0.082

### Differential pTau Pathology and Increased AT8 Immunosignal in PS1 E280A FAD

**FIGURE 2:**
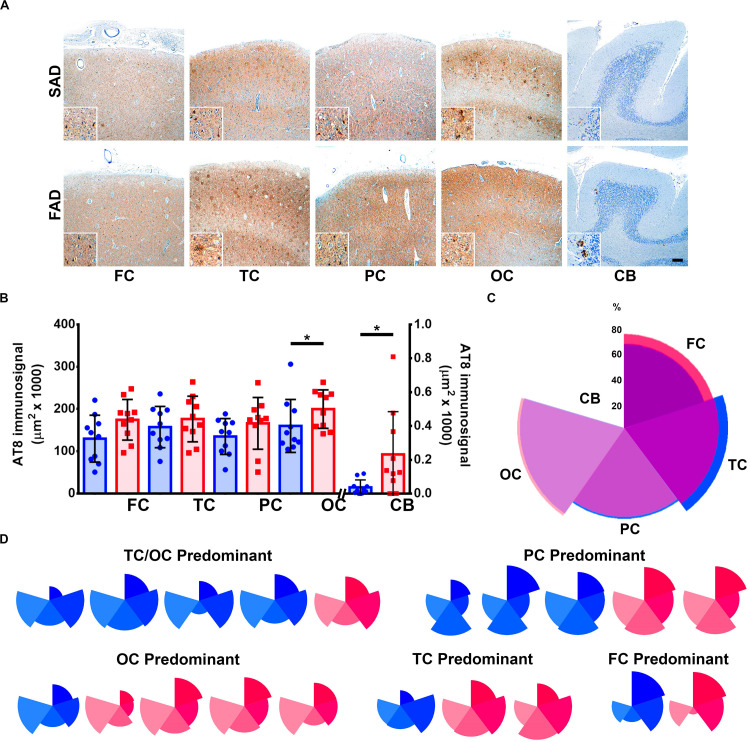
Distribution of Tau pathology brain distribution in SAD vs. FAD PS1 E280A. **(A)** Immunohistochemical staining for pTau (AT8 antibody) in FC, TC, PC, OC, and cerebellum in patients with SAD and FAD (scale bar = 200 mm). Insets depict Tau deposits at higher magnification for each region (scale bar = 70 mm). **(B)** Quantification of pTau immunosignal in both groups. FAD patients showed significantly higher levels in occipital cortex and cerebellum. ^∗^*p* ≤ 0.05. **(C)** Radar chart for the AT8 percentage of immunosignal distribution according to brain regions in SAD (blue) and FAD (red) cases. Both groups present a TC/OC predominant distribution pattern. **(D)** Radar charts for individual AT8 immunosignal distribution pattern for all SAD and FAD cases studied. TC/OC predominant (in which both TC and OC were equally affected) patterns were identified for four SAD and one FAD case. Three PC predominant SAD cases and two FAD cases, one OC predominant SAD case and four FAD cases, one TC predominant case and two FAD cases, and one case of each group presented FC predominant distribution.

It has been previously suggested that Aβ aggregation drives pTau pathology in AD (Stancu et al., 2014). Both SAD and FAD groups presented with abundant pTau AT8 diffuse signal and aggregates (Figure 2A). We found significantly elevated levels of pTau in FAD compared to SAD in occipital cortex, which represented the most affected brain region in both groups. Lowest pTau signals were observed in cerebellum, whereas previously described (Sepulveda-Falla et al., 2011) FAD cases showed significantly more signal than SAD cases (Figure 2B and Table 2). As with Aβ, we analyzed pTau pathology distribution patterns, with average patterns showing a similar predominance of temporal and occipital cortices signal (Figure 2C), Regarding individual patterns, 3/10 SAD and 2/10 FAD cases showed parietal predominance. Four out of 10 SAD and 1/10 FAD cases showed temporo-occipital predominant pattern, while temporal (3/10) and occipital (4/10) predominant patterns were identified in FAD. Interestingly, two FAD cases showed high deposits in all cortices but in the temporal cortex (Figure 2D). Given that general deposition patterns for Aβ and pTau did not show clear differences between SAD and FAD, we analyzed the correlations between both signals for each of the studied regions. Besides the correlation between Aβ in cerebellum and pTau in parietal cortex, no significant correlations between 6E10 and AT8 immunosignals for any other evaluated regions were observed (Supplementary Figure S1 and Supplementary Table S1).

### Characteristic Aβ Species Profile in the Frontal Cortex of PS1 E280A FAD and SAD Patients

**FIGURE 3:**
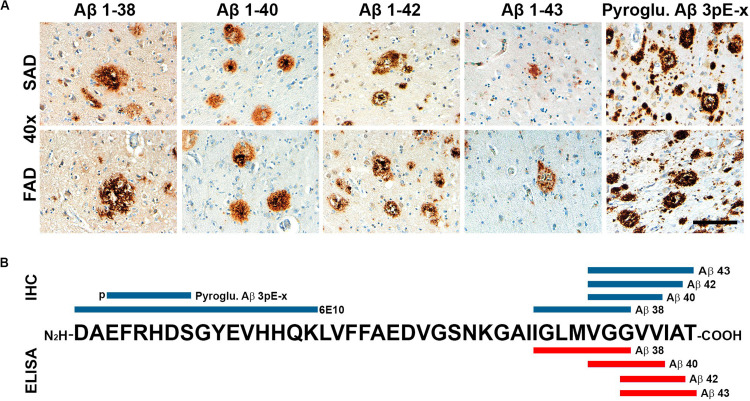
General view of Aβ species evaluated in SAD and FAD cases in this study. **(A)** Immunohistochemical staining in 40 × magnification (scale bar is 100 mm) for Aβ 1-38, Aβ 1-40, Aβ 1-42, and Aβ 1-43. **(B)** Schematic distribution of Aβ-specific epitopes of the antibodies used in this study. Antibodies used for immunohistochemistry (IHC) are in blue and those used for ELISA are portrayed in red.

**FIGURE 4:**
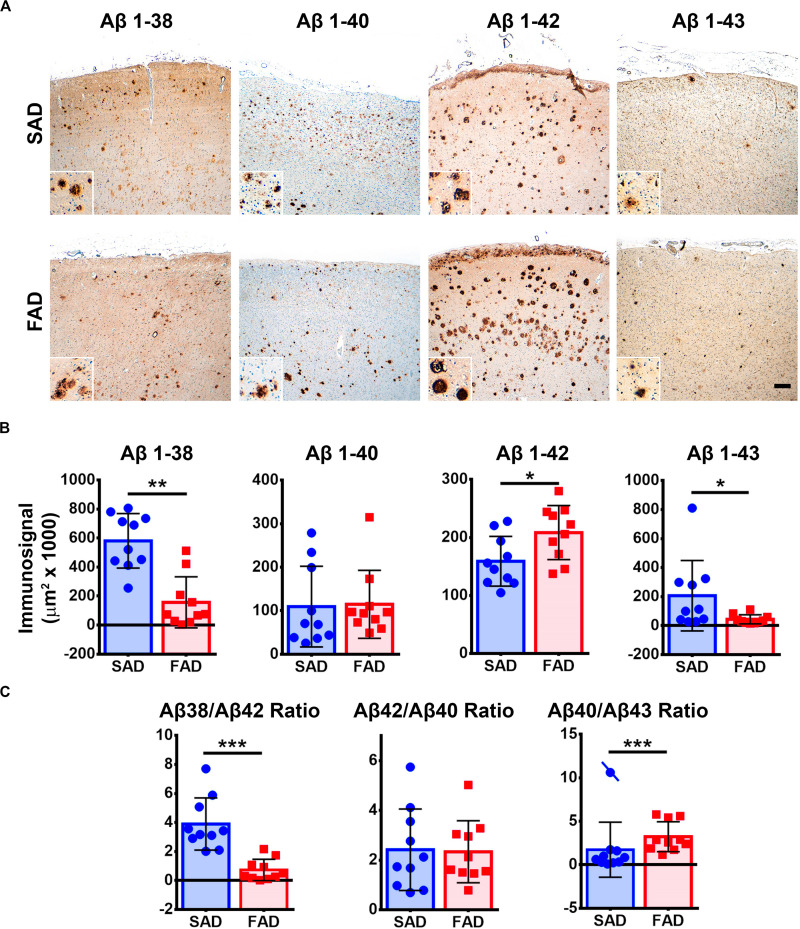
Aβ peptide species pathology profile in SAD and PS1 E280A FAD. **(A)** Immunohistochemical staining for Aβ 1-38, 1-40, 1-42, and 1-43 in FC of SAD and FAD cases (scale bar is 200 mm). Insets depict Aβ deposits at higher magnification (scale bar = 70 mm). **(B)** Quantification of immunosignals for Aβ 1-38, 1-40, 1-42, and 1-43. Significantly higher levels of Aβ 1-38 and 1-43 together with significantly lower levels of Aβ 1-42 were found in SAD when compared to FAD. ^∗^*p* ≤ 0.05; ^∗∗^*p* ≤ 0.01. **(C)** Aβ 1-38/1-42, Aβ 1-42/1-40, and Aβ 1-40/1-43 calculated ratios. SAD cases showed significantly higher Aβ 1-38/1-42 ratio and significantly lower Aβ 1-40/1-43 ratio than FAD cases. Cases crossed by a line are outliers that were not taken in consideration for statistical analysis. ^∗∗^*p* ≤ 0.01; ^∗∗∗^*p* ≤ 0.001.

Given that APP processing and Aβ production are thought to be main features in FAD (Moro et al., 2012), we analyzed deposition of specific Aβ species in frontal cortices of both AD groups (Figures 3A,B). Aβ 1-38 and Aβ 1-43 showed statistically significant higher levels, while Aβ 1-42 showed statistically significant lower levels in SAD compared to FAD. No differences between FAD and SAD were found for Aβ 1-40 (Figures 4A,B). It has been suggested that differences in Aβ ratios can be pathologically relevant (Pauwels et al., 2012). By calculating the Aβ 1-38/1-42, the Aβ 1-42/1-40, and the Aβ 1-40/1-43 ratios, we found significantly higher values in SAD for Aβ 1-38/1-42, and higher values in FAD than in SAD for Aβ 40/43. Aβ 1-42/1-40 ratio showed no difference between both AD groups (Figure 4C).

**FIGURE 5:**
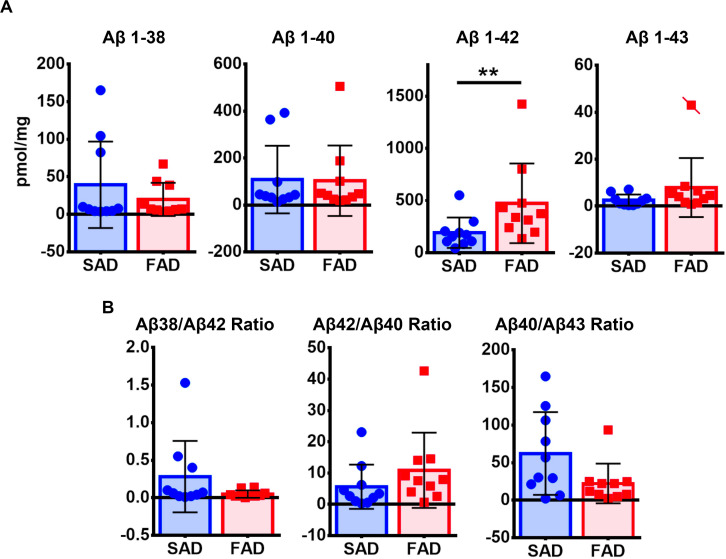
ELISA quantification of Aβ species in SAD and PS1 E280A FAD. **(A)** Aβ1-38, Aβ 1-40, Aβ 1-42, and Aβ 1-43 levels as measured by sandwich enzyme-linked immunosorbent assay in the frontal cortex of SAD and FAD. Only Aβ 1-42 showed significantly higher levels in FAD when compared to SAD. Cases crossed by a line are outliers that were not taken in consideration for statistical analysis. ^∗∗^*p* ≤ 0.01. **(B)** Ratios for Aβ 1-38/1-42, Aβ 1-42/1-40, and Aβ 1-40/1-43 showed no significant differences between both AD groups tested.

Given that IHC staining shows only visible accumulation of Aβ, we performed ELISA for each species (Figure 3B). FAD showed significantly higher amount of Aβ 1-42 in the frontal cortex. In contrast to IHC, the other species (1-38, 1-40, and 1-43) showed no significant differences between groups (Figure 5A). Likewise, there were no significant differences of Aβ ratios in FAD compared to SAD (Figure 5B).

### Characterization of Pyroglutamylated Aβ and CAA Pathology in PS1 E280A FAD

**FIGURE 6:**
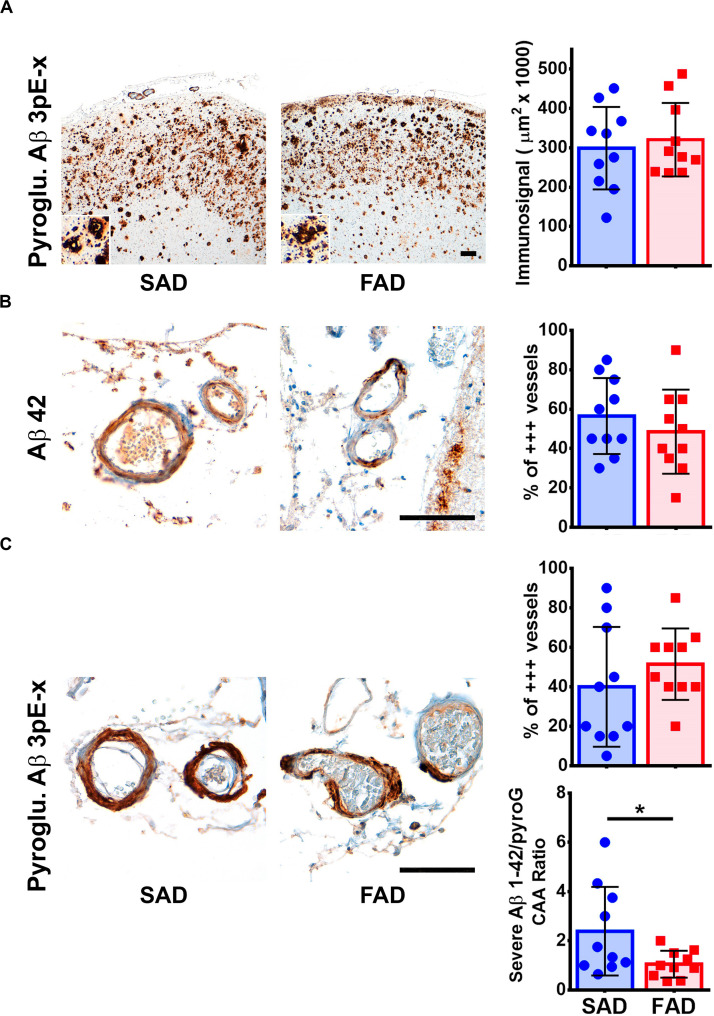
Pyroglutamylated Aβ peptide pathology profile in SAD and PS1 E280A FAD. **(A)** Immunohistochemical staining for Pyroglutamylated Aβ 3pE-x in FC of SAD and FAD patients (scale bar = 200 mm). Insets depict Pyroglutamylated Aβ 3pE-x deposits at higher magnification (scale bar = 70 mm). Quantification of immunosignal for Pyroglutamylated Aβ in both groups. No differences were observed between groups. **(B)** Characterization of Aβ cerebral amyloid angiopathy (CAA) using a specific Aβ 1-42 antibody (as seen in Figure 3). Both, SAD and FAD cases present with severe CAA, with similar percentage of vessels affected in two thirds of their perimeter (% of +++ vessels, *n* = 20). **(C)** Characterization of CAA using a Pyroglutamylated Aβ 3pE-x antibody (as seen in Figure 3). Both SAD and FAD cases present with severe CAA, with similar percentage of vessels affected in two thirds of their perimeter (% of +++ vessels, *n* = 20).

We tested one specific antibody against Pyroglutamylated Aβ 3 to 40-42 (Pyroglu. Aβ 3pE-x) in frontal cortex of SAD and FAD cases. We detected equally strong amyloid pathology in both groups (Figure 6A). The Pyroglu. Aβ 3pE-x antibody showed strong signal in the vessels of both SAD and FAD cases (Figure 6B). Previously, it has been reported that FAD presents with higher CAA scores than SAD (Ringman et al., 2016). We evaluated CAA pathology using Aβ 1-42 and the Pyroglu. Aβ 3pE-x antibodies. We focused on the number of vessels that presented grade 3 CAA pathology assessed according to the Vonsattel grading system (Vonsattel et al., 1991). For both antibodies, there were no statistically significant differences in severe CAA pathology between groups (Figures 6B,C). When an Aβ 1-42/Pyroglu. Aβ 3pE-x ratio was calculated for severe CAA affected vessels, SAD cases presented a statistically significant higher CAA Aβ 1-42/Pyroglu. Aβ 3pE-x ratio when compared to FAD cases (Figure 6C).

## Discussion

Previously we reported specific neuropathological differences between FAD and early onset SAD, with increased 6E10 positive Aβ levels in the frontal cortex and cerebellum of PS1 E280A FAD cases (Sepulveda-Falla et al., 2011), and some Aβ morphological differences in another subset of PS1 E280A patients (Trujillo-Rodríguez et al., 2014). In the present study, we observed no statistically significant differences in 6E10 positive signal from all brain cortices and cerebellum between PS1 E280A FAD cases and similarly, affected SAD cases. It should be kept in mind that 6E10 antibody also stains full length APP containing Aβ domain. However, since antibody positive signal referred specifically to plaques, only pathologic deposits were evaluated. It is possible that early-onset SAD cases might have lower Aβ levels when compared with later-onset SAD (Wolfe, 2007). Both AD groups showed increased Aβ pathology in the frontal cortex, also as a predominant pattern among individuals. This result is in agreement with previous findings regarding Aβ pathology in AD according to PET studies in SAD cases (Barthel et al., 2011) and may reflect region-specific susceptibility toward Aβ pathology (Braak and Del Trecidi, 2011; Thal et al., 2015). It has been reported that Aβ pathology in PS1 FAD is characterized by a diffuse pattern (Shepherd et al., 2009). Our set of severely affected SAD cases showed a similarly, extended diffuse pattern indistinguishable from the observed pattern in FAD when using 6E10A antibody.

Regarding AT8-detected pTau pathology, PS1 E280A FAD showed statistical significance only in the occipital cortex and cerebellum; this last feature was as previously reported (Sepulveda-Falla et al., 2011). On an individual basis, predominantly affected areas differ between SAD and FAD, suggesting a differential pTau pathological process between FAD and SAD (Sepulveda-Falla et al., 2011; Trujillo-Rodríguez et al., 2014). PS1 mutations affect the activation of kinase cascades (Baki et al., 2008), which could explain this difference. Finally, the lack of correlation between Aβ and pTau pathology in both FAD and SAD confirms previous findings about independent pathology processes in AD (Braak and Del Trecidi, 2011). It has been suggested that in SAD, pTau pathology could precede Aβ plaque deposition. Given that FAD is caused by γ secretase dysfunction, Aβ pathology should precede pTau pathology; the fact that pTau aggregation patterns show no association with the Aβ aggregation pattern suggests that other events independent of Aβ production may be involved in FAD.

Presenilin-1 mutations affect γ secretase activity, shifting the type of Aβ peptides generated. An alternating forked model for the successive epsilon cut of C99 APP fragment has been proposed, in which γ secretase generates either Aβ 1-46 > 1-43 > 1-40 or Aβ 1-45 > 1-42 > 1-38 peptides (Chávez-Gutiérrez et al., 2012; Golde et al., 2013). It has been shown in PS1 E280A cellular models that the Aβ 1-42/1-40 ratio is decreased, by decreasing Aβ-1-42 to Aβ 1-38 turnover (Li et al., 2016). We recently characterized Aβ peptide production by purified gamma secretase from postmortem brain tissue from several pS1 mutations. PS1 E280A showed decreased Aβ 1-38, Aβ 1-40, and total Aβ, together with increased Aβ 1-42/Aβ 1-40 ratio and decreased Aβ 1-38/Aβ 1-42 ratio when compared to SAD and controls (Szaruga et al., 2017).

For analyzing morphology and distribution of Aβ 1-38, 1-40, and 1-42 peptides, we used a set of antibodies developed by Janssen R&D [43] and characterized by high specificity and sensitivity. We identified a clear and complementary difference of Aβ 1-38 and Aβ 1-42 immunosignals in frontal cortex between FAD and SAD cases, suggesting that mutated PS1 favors Aβ 1-42 over Aβ 1-38 production, confirming previous findings (Szaruga et al., 2017). Even though increased Aβ 1-42 immunosignal in this population (Lemere et al., 1996) and in FAD in general (Kumar-Singh et al., 2006) has been widely reported, decreased 1-38 signal is a novel neuropathological finding in agreement with altered γ secretase function as described *in vitro* for PS1 FAD (Chávez-Gutiérrez et al., 2012; Szaruga et al., 2017) and in contrast to a recent report studying 1-38 pathology in APP, PS1, and PS2 FAD cases comparing them with SAD. In that report, neither PS1 mutant carriers nor SAD cases without CAA presented with Aβ 1-38 pathology. The PS1 E280A mutation was not included (Moro et al., 2012). In our study all severely affected SAD cases showed higher Aβ 1-38 loads when compared to PS1 E280A FAD. The differences between studies could be explained by the severity of studied SAD patients and specificity/sensitivity of the antibodies used.

Regarding Aβ 1-43 and 1-40 immunosignal, only Aβ 1-43 showed higher levels in SAD. Previous reports in human brain tissue indicated Aβ 1-43 to be more frequently observed in the core of Aβ plaques (Welander et al., 2009) and to accumulate more than Aβ 1-40 in SAD brains (Saito et al., 2011). It is to be noted that in the first study only one case of PS1 FAD was investigated. Furthermore, by evaluating Aβ 1-38/1-42 and Aβ 1-40/1-43 ratios, we confirm cell culture-based studies suggesting FAD-specific Aβ production (Chávez-Gutiérrez et al., 2012). Higher levels of Aβ 1-38 and Aβ 1-43 are favored in SAD while Aβ 1-42 production is specifically increased in FAD. Interestingly, the Aβ 1-42/1-40 ratio showed no difference between FAD and SAD cases, which is in opposition to previous findings (Kumar-Singh et al., 2006). This ratio could vary depending on PS1 mutation and on AD pathology severity of the SAD cases studied. Recently, the pathogenicity of Aβ 1-43 in FAD has come to the fore. It has been described that increased production of Aβ 1-43 in specific PS1 FAD mutations is a reflection of increased γ secretase dysfunction (Trambauer et al., 2020). Perhaps low Aβ 1-43 levels detected in our FAD samples are related with the relatively mild γ secretase dysfunction observed in PS1 E280A brain homogenates (Szaruga et al., 2015).

One possible objection for morphological differences in Aβ species detection could be that observed changes represent the endpoint of Aβ pathology (Serrano-Pozo et al., 2012). Thus, we evaluated Aβ species by ELISA. Aβ 1-38, 1-40, and 1-42 results resemble our histological findings. None of the calculated Aβ ratios (as measured by ELISA) were significantly different as opposed to histological findings and in contrast to some of our previous results obtained with less severely affected SAD cases (Sepulveda-Falla et al., 2012). Furthermore, Aβ as detected by ELISA may not be identical to Aβ detected by *in situ* methods. Aβ deposits can reflect long-term Aβ pathology better because only a continuous phenomenon could generate them, whether by increased production or impaired degradation. We propose that Aβ 1-38/1-42 and Aβ 1-40/1-43 ratios as assessed by immunohistochemistry are a sensitive enough tool for the neuropathological study of both AD groups. Our study shows Aβ species profile in only one PS1 mutation. Other PS1 mutations could present with different Aβ species profiles. However, among PS1 mutations, there are common features such as Aβ 1-38/1-42 ratio (Li et al., 2016; Szaruga et al., 2017) that could result in similar IHC findings.

Post-translational modifications of Aβ, like pyroglutamylated and truncated forms, deposit alongside other Aβ species (Cabrera et al., 2018). Pyroglu. Aβ 3pE-x have been identified as depositing early in humans and primates while depositing later in transgenic mouse models (Frost et al., 2013). It seems to be specifically associated with AD pathology in contrast to age-associated Aβ deposits (Moro et al., 2018), or to pure CAA (Gkanatsiou et al., 2019). In our analysis, both severely affected SAD and PS1 E280A FAD frontal cortices showed equally extensive Pyroglu. Aβ 3pE-x plaque and vessel pathology. However, Pyroglu. Aβ 3pE-x antibody detected relatively more severely affected vessels in FAD than those detected with Aβ 1-42 antibody. This difference could be related with the temporal order in which different Aβ species are deposited in SAD and FAD. For FAD, higher Aβ 1-42 generation is constant throughout life, while Pyroglu. Aβ 3pE-x deposits can be associated with aging (Moro et al., 2018). Further studies should assess if truncated Aβ species play a larger role in CAA in PS1 FAD. Besides, vascular impact of Pyroglu. Aβ 3pE-x aggregation can be of relevance given the possible use of pyroglutamylated Aβ detection in plasma as a biomarker for plaque pathology in AD (Wang et al., 2020).

We have previously reported neuropathological differences between PS1 E280A FAD and SAD cases, including differential pTau pathology (Sepulveda-Falla et al., 2011), distinct APP processing patterns (Pera et al., 2013), and mitochondrial damage and decreased Ca^2+^ channels in the cerebellum (Sepulveda-Falla et al., 2014). In this study, we describe yet another difference between PS1 E280A FAD and SAD, regarding Aβ pathology and possibly linked to the underlying γ secretase dysfunction in PS1 FAD cases. We propose the usage of specific Aβ 1-38, 1-40, 1-42, and 1-43 antibodies and their immunosignal values and ratios as a viable option for better assessment of FAD cases and the identification of SAD cases with similarly, altered Aβ production to be studied further.

## Data Availability Statement

The raw data supporting the conclusions of this article can be made available by the corresponding author upon reasonable request.

## Ethics Statement

The studies involving human participants were reviewed and approved by Comité de Bioética, Facultad de Medicina, Universidad de Antioquia. Acta número 011 de 2016. The patients/participants provided their written informed consent to participate in this study.

## Author Contributions

FD, DT-R, and DS-F performed the experiments. FD, JS, MM, MG, and DS-F set up the experimental design. FD, AV, DT-R, FL, and DS-F were involved in data and or patient material acquisition. FD, MG, and DS-F wrote the manuscript and was revised by all authors.

## Conflict of Interest

The authors declare that the research was conducted in the absence of any commercial or financial relationships that could be construed as a potential conflict of interest.
