# Supplementary material for: Decreased Deposition of Beta-Amyloid 1-38 and Increased Deposition of Beta-Amyloid 1-42 in Brain Tissue of Presenilin-1 E280A Familial Alzheimer’s Disease Patients
Source: Front Aging Neurosci. 2020 Jul 28;12:220. doi: 10.3389/fnagi.2020.00220 (PMC7399638; doi:10.3389/fnagi.2020.00220)
Supplement: Supplementary file 1 [file Data_Sheet_1.pdf]

Supplementary Material for:

Decreased Deposition of Beta-Amyloid 1-38 and increased Deposition of Beta-Amyloid 1-42 in Brain Tissue of Presenilin1 E280A Familial Alzheimer's Disease Patients

Felix Dinkel[1]; Diana Trujillo-Rodriguez MSc[2], Andres Villegas, MD, PhD[3]; Johannes Streffer, MD[4]; Marc Mercken, PhD[4], Francisco Lopera, MD[3]; Markus Glatzel, MD[1*], Diego Sepulveda-Falla, MD[1*¥]

1. Institute of Neuropathology, University Medical Center Hamburg-Eppendorf - UKE, Hamburg, Germany.
2. Institute of Genetics, National University of Colombia, Bogotá, Colombia.
3. Neuroscience Group of Antioquia, Faculty of Medicine, University of Antioquia, Medellín, Colombia.
4. J&J Pharmaceutical Research and Development, Janssen Pharmaceutica, Beerse, Belgium

* These authors contributed equally

¥ Correspondence to: Diego Sepulveda-Falla, Institut für Neuropathologie, Universitätsklinikum Hamburg-Eppendorf, Martinistraße 52, 20246, Hamburg, Germany. E-mail: dsepulve@uke.de. Phone: +49 40 741051920, Fax: +49 40 741055696

Supplementary Table 1

	Variable	SAD	PS1-E280A FAD
**Demographics**	**Age of Onset**		(-) ELISA Aβ 40/43
	**Brain Weight**		(-) Age of Death, (-) Disease Duration, (+) IHC pTau FC, (+) IHC Aβ 38 FC, (+) IHC 38/42, (+) ELISA Aβ 1-40, (-) ELISA Aβ 42/40, (+) ELISA Aβ 1-38, (+) ELISA Aβ 38/42
	**Age of Death**	(+) IHC 6E10 FC	(-) IHC pTau FC, (-) ELISA Aβ 1-40, (+) ELISA Aβ 42/40, (-) ELISA Aβ 1-38, (-) ELISA Aβ 38/42
	**Disease Duration**		(-) IHC pTau FC, (-) IHC PyroG Aβ FC, (-) ELISA Aβ 1-40, (-) ELISA Aβ 1-43, (-) ELISA Aβ 1-38, (-) ELISA Aβ 38/42
**6E10 IHC**	**Frontal Cortex**	(+) IHC 6E10 OC	(+) IHC 6E10 TC, (+) ELISA Aβ 1-38
	**Temporal Cortex**	(+) IHC Aβ 1-40 FC, (-) IHC 42/40 FC	
	**Parietal Cortex**		
	**Occipital Cortex**	*(+) IHC Aβ 1-42 FC*, (+) ELISA Aβ 1-38	*(+) IHC Aβ 1-42 FC*, (-) IHC Aβ 43 FC, (+) IHC Aβ 40/43, (+) CAA 1-42
	**Cerebellum**	(+) ELISA Aβ 38/42	(+) IHC pTau PC, (+) IHC Aβ 1-40 FC, (-) IHC Aβ 42/40 FC
**pTau IHC**	**Frontal Cortex**	*(+) IHC 40/43 FC*	*(+) IHC 40/43 FC*, (+) ELISA Aβ 1-40, (+) ELISA Aβ 1-38, (+) ELISA Aβ 38/42, (+) CAA 1-42
	**Temporal Cortex**	(+) IHC pTau OC, (+) IHC Aβ 43 FC, (-) ELISA Aβ 1-38, (-) CAA 1-42	(-) IHC Aβ 42/40 FC, (+) IHC Aβ 38/42 FC
	**Parietal Cortex**	(+) Aβ 38 FC, (-) Ratio 40/43 FC	(+) IHC PyroG Aβ FC
	**Occipital Cortex**		(+) IHC Aβ 1-40 FC
	**Cerebellum**	(+) IHC Aβ 1-42 FC	
**Aβ Antibodies IHC**	**Aβ 40 FC**	(+) ELISA Aβ 1-40, (+) ELISA Aβ 1-38, (+) CAA PyroG	
	**Aβ 42 FC**	(+) ELISA Aβ 1-38, (+) CAA PyroG	(+) IHC Aβ 40/43 FC
	**Aβ 38 FC**		(+) ELISA Aβ 38/42
	**Aβ 43 FC**		(-) IHC Aβ 42/40 FC, (-) CAA 1-42
	**Ratio 42/40 FC**	(-) ELISA Aβ 1-40, (-) ELISA Aβ 1-38, (-) CAA PyroG	
	**Ratio 38/42 FC**		(-) ELISA Aβ 42/40, (+) ELISA Aβ 40/43, (+) ELISA Aβ 38/42
	**Ratio 40/43 FC**		(+) CAA 1-42, (+) CAA PyroG
	**PyroG Aβ FC**		
**Aβ ELISA**	**Aβ 1-40**	*(+) ELISA Aβ 1-38*, (+) ELISA Aβ 38/42, (+) CAA 1-42, (+) CAA PyroG	(+) ELISA Aβ 1-43, (+) *ELISA Aβ 1-38*
	**Aβ 1-42**	(+) ELISA Aβ 1-43	
	**Aβ 42/40**	(-) ELISA Aβ 40/43, *(-) ELISA Aβ 38/42*, (-) CAA PyroG	*(-) ELISA Aβ 38/42*
	**Aβ 1-43**		(+) ELISA Aβ 1-38
	**Aβ 40/43**	(+) ELISA Aβ 38/42, (+) CAA PyroG	
	**Aβ 1-38**	(+) CAA 1-42, (+) CAA PyroG	
	**Aβ 38/42**	(+) CAA 1-42, (+) CAA PyroG	
**CA**	**Aβ 1-42**		
	**Aβ PyroG**		

(-) = Negative correlation, Aβ = Amyloid beta, (+) = Positive correlation, IHC= Immunohistochemistry, pTau = hyperphosphorylated Tau, FC = Frontal cortex, TC = Temporal cortex, PC = Parietal cortex, PyroG = Pyroglutaminated Aβ, CAA = Cerebral Amyloid Angiopathy.

Supplementary Figure 1

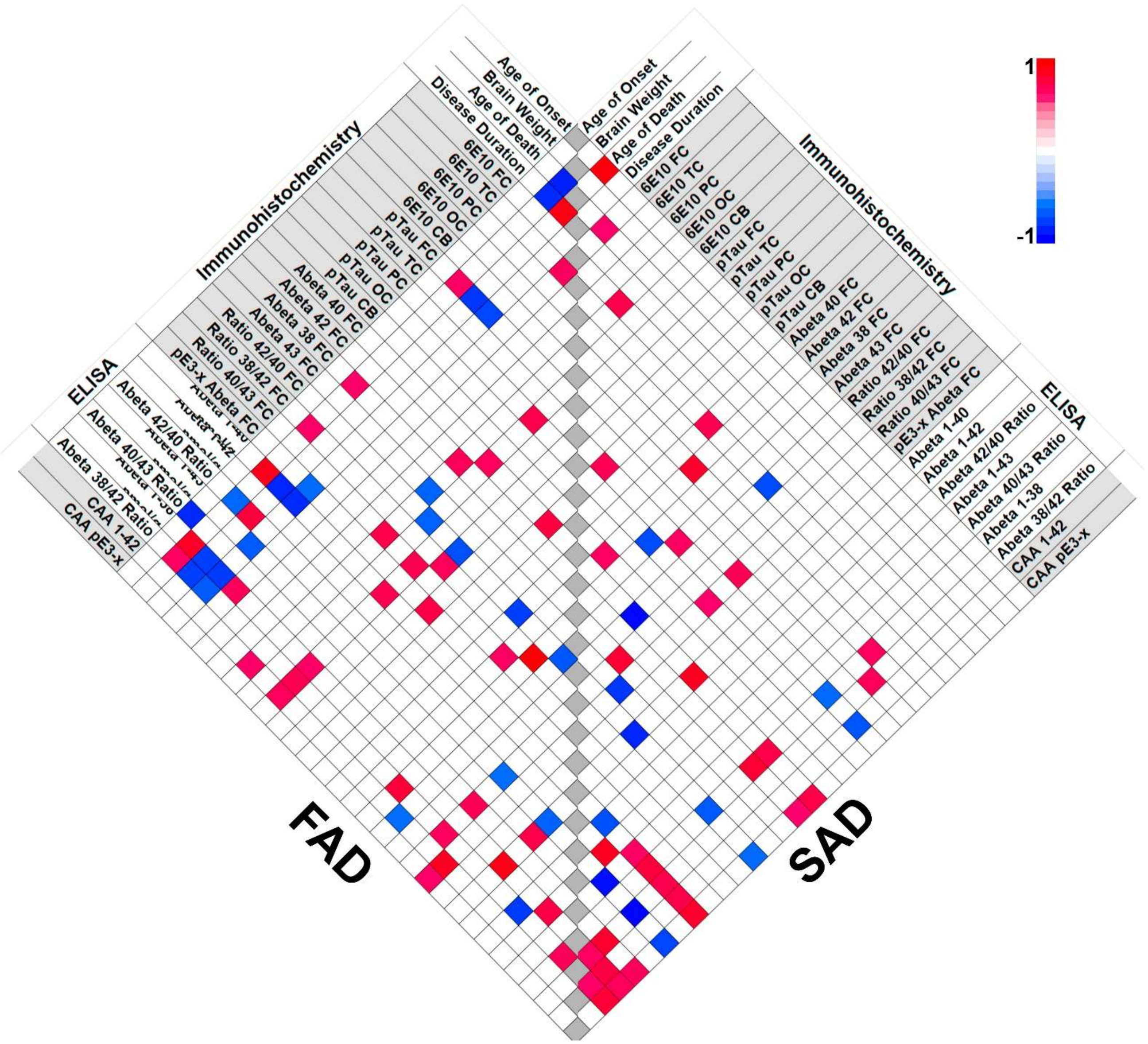

Supp. Fig. 1. Heatmap depicting statistically significant correlations between analyzed variables in this study. Positive Spearman's ρ values are depicted in red and negative values in blue.
